# Disclosing the production of fungal chitinases and proteases using insect by‐products as substrates in solid‐state fermentation

**DOI:** 10.1002/jsfa.70912

**Published:** 2026-08-02

**Authors:** Borja Fernández, Helena Fernandes, Nuno Muñoz‐Seijas, Francisco Soto‐Beltrán, José Manuel Domínguez, Helena Peres, José Manuel Salgado

**Affiliations:** ^1^ University of Vigo, Campus Auga, Industrial Biotechnology and Environmental Engineering Group ‘BiotecnIA’ Ourense Spain; ^2^ Interdisciplinary Centre of Marine and Environmental Research (CIIMAR) Terminal de Cruzeiros Do Porto de Leixões, Universidade Do Porto Matosinhos Portugal; ^3^ Laboratorio Cifga Lugo Spain; ^4^ Department of Biology, Faculty of Sciences University of Porto Porto Portugal

**Keywords:** adult insects, chitinases, frass, insect by‐products, proteases, solid‐state fermentation

## Abstract

**BACKGROUND:**

The world population is projected to reach nearly 9.7 billion by 2050, significantly increasing the demand for novel and sustainable protein sources. Edible insects, such as *Tenebrio molitor*, emerge as promising alternatives due to their high nutritional value, but adult insects and frass are direct and abundant by‐products of this industry. This study aimed to valorize insect by‐products through solid‐state fermentation (SSF) to produce chitinases and proteases with high industrial value.

**RESULTS:**

Various fungi were tested in SSF, with *Phanerochaete chrysosporium* CECT 2777 and *Aspergillus uvarum* MUM 08.01 standing out as the most efficient protease producers, while *Aspergillus niger* CECT 2088 showed higher chitinolytic activity. Maximum chitinase (53.3 U g^−1^) and protease (366 U g^−1^) production was achieved on the 11th and 9th day of SSF of frass and adult insects, respectively, allowing the optimization of fermentation time. Increasing the solid load in SSF up to 50 g dry weight (DW) of frass did not decrease chitinase production (47.8 U g^−1^) compared to using 10 g DW, while protease activity decreased 70% and 44% when the quantity of adult insects increased up to 50 g DW and 100 g DW, respectively.

**CONCLUSION:**

This work is pioneering in demonstrating the great potential of reusing *Tenebrio molitor* by‐products to obtain value‐added chitinases and proteases through SSF, contributing to reducing waste accumulation of insect origin and promoting the development of a circular economy. © 2026 The Author(s). *Journal of the Science of Food and Agriculture* published by John Wiley & Sons Ltd on behalf of Society of Chemical Industry.

## INTRODUCTION

The global population is projected to reach 9.7 billion by 2050, requiring a 70% increase in food production^1^ and increasing the demand for other fundamental resources such as water and energy.^2^ This scenario concomitantly increases the pressure on ecosystem services, boosting efforts to reduce environmental pollution, avoiding the disposal of by‐products and evaluating novel processes for obtaining valuable compounds.^3^

In recent years, insect farming has been gaining popularity as a sustainable alternative to traditional proteins, decreasing dependence on livestock production and its associated environmental impacts.^4^, ^5^ In 2019, it was estimated that nearly 250 companies worldwide were working with insects for food and feed,^2^ and more recent prospects indicate that insect production will increase from 500 tons in 2019 up to 260 000 tons by 2030.^6^ The success of the insect industry is related to the high content of quality protein, presence of valuable biocompounds, and minimal environmental impact, mainly due to high feed efficiency, low requirements in water, energy and space, and low or no emission of greenhouse gases.^7^, ^8^, ^9^

Among the edible insects authorized for human consumption in the European Union (Regulation 2015/2283), *Tenebrio molitor* larvae (commonly named mealworms) are one of the most promising, and already regulated by the European Food Safety Agency (EFSA).^10^ Mealworms are a good source of protein (48.5–50% on a dry weight [DW] basis), lipids (13–30.5% DW), highlighting the polyunsaturated fatty acid and micronutrient content.^11^, ^12^ Insects may also be reared using various organic by‐products as feeding sources (fermented corn straw, brewer's bagasse, wheat bran, wine bagasse, among others), improving the circular economy and sustainability of this industry.^4^, ^13^ Nonetheless, since only the larvae are authorized in foods and feeds, the adult insects (commonly named beetles) are directly disposed of, since the high chitin content hinders the bioaccessibility of protein.

Another abundant by‐product from the insect industry is frass (2–3 kg generated per kilogram of mealworms), in which 1.5 million tons of frass were expected to be generated each year until 2025.^3^, ^5^, ^9^, ^14^ Frass is composed of insect feces, uneaten food (mostly lignocellulosic materials), and cuticles shed from the exoskeletons of growing larvae (mainly composed of chitin)^5^, ^9^ and used as a by‐product in animal feed^9^ and as a fertilizer,^2^ due to the content in bioactive compounds.^15^ However, with the prospects of increasing insect production, frass disposal is expected to increase. In this sense, frass may be valorized through fermentative processes, such as solid‐state fermentation (SSF). SSF may be used to produce a wide range of compounds, including enzymes, allowing reusing agro‐industrial and food‐derived by‐products as substrates for microbial growth, reducing production costs and often resulting in high production efficiencies and product recovery.^16^

Microbial enzymes are widely used in different industries such as in textile, pharmaceutical, chemical engineering, and food technology fields, among others.^17^, ^18^ The most explored enzymes are proteases, chitinases, cellulases, and xylanases, which may be produced from animals, plants, microorganisms, and fungi.^12^ Proteases (EC 3.4.21–24) can be classified as acidic, neutral, or alkaline, depending on their optimal pH range, representing up to 65% of the global enzyme market, with annual sales ranging between USD 1.5 and 1.8 billion.^19^, ^20^, ^21^ These enzymes can hydrolyze food or feed ingredients, including insect meals, obtaining proteins with enhanced bio‐ and/or techno‐functionalities.^22^ In turn, chitinases (EC 3.2.1.14) hydrolyze insoluble chitin biopolymers into their monomeric and oligomeric components, breaking the β‐1,4‐glycosidic bonds of *N*‐acetyl‐d‐glucosamine.^17^, ^23^ Among their main functions, chitinases are involved in insect molting, digestion, and pathogenicity,^23^ and as a defensive mechanism against fungal pathogens in plants.^17^ In fungi, chitinases play an important role in growth, regulating the hyphal growth, division, and branching of these microorganisms.^24^ Therefore, it is expected that, besides the chitin‐rich composition of fungal cell walls, the presence of a substrate or mixture of substrates rich in chitin during SSF would promote and/or potentiate the production of chitinases. Indeed, different studies indicate that mixing substrates improves chitinase production, probably due to higher nutrient diversity and availability for fungal growth.

In this sense, achieving efficient, sustainable and low‐cost production of proteases and chitinases is of utmost importance due to their wide‐ranging applications and economic value, such as in pharmaceutical, food, and biotechnology industries.^25^ Currently, enzymes are mainly produced by submerged fermentation (SmF) but other methods such as SSF may be considered that allow the use of undervalued and abundantly disposable substrates for microbial growth.^24^ In a previous study, Muñoz‐Seijas *et al*. (2024)^12^ observed that SSF of frass with *Aspergillus uvarum* MUM 08.01 resulted in high protease activity, and that mixing frass with brewer's spent grain increased protease production. Even so, besides proteases, insect by‐products may also be used to induce chitinase production given the chitin content of these biomasses, while other fungal species may also be studied besides the *Aspergillus* genus, such as *Neurospora crassa* and *Phanerochaete chrysosporium*, among others, given their chitinase synthesis capability.^26^, ^27^ Therefore, this work aims to produce proteases and chitinases via SSF using the main by‐products of *Tenebrio molitor* production – frass and adult insects – as substrates. For this purpose, different fungal species were first tested in SSF to determine the most efficient enzyme producer. An experimental design was also conducted to assess whether mixing frass with adult insects and/or chitin of insect origin would improve enzyme production, aiming to achieve an integrated reutilization of both residues. After selecting the most suitable fungal strain and substrate for protease and chitinase production, SSFs were performed using higher substrate loads, foreseeing the production of high quantities of both enzymes to envision a feasible application of SSF in insect by‐products.

## MATERIALS AND METHODS

### Raw materials and microorganisms

*Tenebrio molitor* adult insects (initial moisture content of 16.30% ± 0.24%) and frass (initial moisture content of 8.05% ± 0.25%), provided by TecmaFoods (Leça do Balio, Portugal), were finely ground (particle size ≤0.5 mm; Sogo Grinder, 300 W) and storYeed at room temperature until use. The chitin used in the simplex centroid design was obtained from Muñoz‐Seijas *et al*.,^28^ after submitting *Tenebrio molitor* adults to a treatment using deep eutectic solvents (DES; composed of betaine and urea, 1:2 molar ratio, 30% distilled water, at 90 °C, 3 h). The Spanish Type Culture Collection (CECT; Valencia, Spain) provided different fungal species used in SSF of insect by‐products (Supplementary Material): *Aspergillus niger* (CECT 2088), since *Aspergillus* spp. show high capability to produce a wide range of enzymes, including proteases;^29^ *Mucor circinelloides* (CECT 265), for its ability to produce extracellular proteases;^30^ *N. crassa* (CECT 2727), capable of expressing chitinase‐related genes;^19^ *Acremonium chrysogenum (*CECT 2723), since *Acremonium* spp. demonstrated chitinolytic capacity;^31^ *P. chrysosporium* (CECT 2777), possessing genes related to lignocellulose‐degrading enzymes and chitinases;^32^ and *Agaricus bisporus* (CECT 20291), to assess whether it would be possible to grow this edible mushroom using insect by‐products while simultaneously producing valuable enzymes. *Aspergillus uvarum* MUM 08.01 was provided by the Micoteca of the University of Minho (MUM; Braga, Portugal).

### Screening of different fungi for chitinase and protease production by SSF

*Aspergillus niger* CECT 2088, *Mucor circinelloides* CECT 2655, *Acremonium chrysogenum* CECT 2723, *P. chrysosporium* CECT 2777, *Agaricus bisporus* CECT 20291 and *Aspergillus uvarum* MUM 08.01 were grown in a culture media of potato dextrose agar (PDA; 300 g L^−1^ diced potatoes, 20 g L^−1^ glucose, 15 g L^−1^ agar powder). *Neurospora crassa* CECT 2727 was grown in Winge medium (3 g L^−1^ yeast extract, 20 g L^−1^ glucose, 15 g L^−1^ agar power). All culture media were sterilized (121 °C, 15 min) before the inoculation of each fungus.

Initially, the screening of the different fungal strains for SSF of the insect by‐products was performed using 10 g dry weight (DW) of frass in 250 mL Erlenmeyer flasks. The moisture content was adjusted to 750 g kg^−1^ with distilled water and sterilized (121 °C, 15 min). The moisture content was defined based on previous works observing high enzymatic activities using fundi in SSF (Muñoz‐Seijas *et al*., 2024)^12^. The flasks were then cooled at room temperature and inoculated with 2 × 10^5^ spores of *Aspergillus niger* CECT 2088 or *Aspergillus uvarum* MUM 08.01 per gram of dry frass. The spores were suspended in a spores' solution (1 g kg^−1^ peptone and 0.1 g kg^−1^ Tween 80). Since the other fungal species (*Mucor circinelloides* CECT 2655, *Acremonium chrysogenum* CECT 2723, *P. chrysosporium* CECT 2777, *Agaricus bisporus* CECT 20291, *N. crassa* CECT 2727) did not sporulate, each flask was inoculated with five disks of 1 cm diameter.^33^ All flasks were incubated at 25 °C for 7 days in a moisturized environment (VWR IL150R Premium incubator, Avantor; 50% fan intensity). The SSF using each fungal strain was carried out in triplicate.

At the end of SSF, the enzymes were extracted according to Fernandes *et al*. (2022)^34^, mixing the total quantity of the solid‐fermented substrate of each Erlenmeyer flask with distilled water (solid‐to‐liquid ratio of 1:5, w/v), for 30 min at room temperature, and with constant agitation (150 rpm). The mixtures were centrifuged (2021 × *g*, 15 min, 4 °C), vacuum‐filtrated through filter paper (0.2 μm pore size; Whatman No. 1) and the liquid was stored at −20 °C pending determination of enzyme activities.

After assessing the most suitable enzymes producer (*Aspergillus niger* CECT 2088), the adult insects were also used as substrate in SSF to assess protease production, following the methodology described.

### Kinetics of enzyme production in SSF of frass and adult insects

Frass and adult insects were solid fermented with *Aspergillus niger* CECT 2088 for 10 and 14 days, respectively, following the procedure described in the section ‘Screening of different fungi for chitinase and protease production by SSF’, above. Triplicate samples were collected at specific sampling times during SSF: that is, on days 2, 4, 7, 9, 10, 11, 12, and 14 in SSF of frass, and on days 2, 4, 7, 9, and 10 in SSF of adult insects. Three Erlenmeyer flasks were removed and the entire fermented substrate of each flask was mixed with distilled water (solid‐to‐liquid ratio of 1:5, w/v), for 30 min, at room temperature, and constant agitation (150 rpm) to extract the enzymes. The samples were centrifuged (2021 × *g*, 15 min, 4 °C) and vacuum‐filtrated through filter paper (0.2 μm pore size; Whatman No. 1); the liquid fractions were stored at −20 °C pending assessment of the enzymatic activities.

### Optimization of substrate composition for protease and chitinase production by SSF

A simplex centroid mixture design was carried out to assess possible synergetic effects of combining different substrates – frass, adult insects and chitin (obtained after processing adult insects with DES^28^ – in the production of fungal proteases and chitinases. This design consisted of nine experiments with each independent variable (frass, adult insects, chitin) at three levels: 1 (100%), 1/2 (50%), and 1/3 (33%), fixing the final amount of substrate in 10 g DW. The SSFs and enzyme extractions were carried out following the methods described in the section ‘Screening of different fungi for chitinase and protease production by SSF’, above. The enzymatic extracts were stored at – 20 °C pending assessment of chitinase and protease activities.

By applying multiple regression analysis on the experimental data, the following equation representing this model was obtained:

$$Y=b_{1}x_{1}+b_{2}x_{2}+b_{3}x_{3}+b_{12}x_{1}x_{2}+b_{13}x_{1}x_{3}+b_{23}x_{2}x_{3}+b_{123}x_{1}x_{2}x_{3}$$

where *Y* is the response variable (chitinases or proteases), *b* are the coefficients of regression, and *x* are the independent variables.

### Tray reactor experiments in the SSF for chitinase and protease production

The quantity of frass and adult insects used in SSF for chitinase and protease production, respectively, was increased, and the SSFs were carried out in trays measuring 22 × 16.5 × 7.5 cm (with 50 g DW of substrate) or 35 × 24 × 7 cm (with 100 g DW of substrate). In each tray, the bed height was adjusted to 2.5 cm and moisture was adjusted to 750 g kg^−1^ with distilled water, followed by sterilization at 121 °C, for 15 min. Each tray was inoculated with 2 × 10^5^ spores of *Aspergillus niger* CECT 2088 per gram of dried substrate, covered with perforated plastic wrap to avoid contamination while ensuring air exchange and avoid overheating during SSF, and incubated at 25 °C (VWR IL150R Premium incubator, Avantor; 50% fan intensity), for 11 (frass) or 9 (adult insects) days, for chitinase and protease production, respectively.

### Determination of enzyme activity

Chitinase activity (EC 3.2.1.14) was measured using 4‐nitrophenyl *N*‐acetyl‐β‐d‐glucosaminide; 1 mmol L^−1^, pH 6) in 0.2 mol L^−1^ sodium phosphate buffer (pH 6) as substrate, following Alves *et al*.,^35^ with some modifications. Briefly, 100 μL of sample was mixed with 100 μL of substrate and incubated at 37 °C, for 15 min. Then, 1 mL of 1 mol L^−1^ NaOH was added to stop the reaction, 300 μL of each sample was transferred to a well of a 96‐well microplate, and absorbance was read at 405 nm. The chitinolytic activity was quantified using 4‐nitrophenol standard at concentrations ranging between 0.013 and 0.2 g L^−1^. A blank of each sample was used, deactivating the enzymes by boiling the samples at 100 °C for 5 min. One unit of chitinase activity was defined as the amount of enzyme necessary to increase 1 μmol of product released per minute per gram of dried and solid‐fermented substrate. The results were expressed as activity units per gram of dried and solid‐fermented substrate (U g^−1^).

Acid protease activity was determined following the method of Sousa *et al*.,^36^ using azocasein (5 g L^−1^) dissolved in 50 mmol L^−1^ sodium acetate buffer (pH 5) as substrate. Briefly, 500 μL of each sample was mixed with 500 μL of substrate and incubated at 37 °C, for 40 min. Then, 1 mL of trichloroacetic acid (100 g L^−1^) was added and the mixture was centrifuged (2021 × *g*, 15 min, at 4 °C). The supernatants were mixed with 5 mol L^−1^ KOH solution, and the absorbance was read at 450 nm. A blank of each sample was prepared by inactivating the enzymes (100 °C, 5 min). One unit of protease activity was defined as the amount of enzyme required to produce a 0.01% increase in absorbance relative to the respective blank at 37 °C, pH 5, per minute. Protease activity was expressed in units per gram of dried and solid‐fermented substrate (U g^−1^).

Cellulase and xylanase activities were quantified in the enzymatic extracts obtained in the screening of fungal strains, using carboxymethylcellulose (20 g L^−1^ in 0.05 mol L^−1^ sodium citrate buffer, pH 4.8) and xylan (10 g L^−1^ in 0.05 mol L^−1^ sodium citrate buffer, pH 4.8) as substrates, respectively. Samples were incubated at 50 °C for 15 min (xylanases) or 30 min (cellulases), followed by assessing the free sugars released using the 3,5‐dinitrobenzoic acid method.^37^ One unit of cellulase or xylanase activity was defined as the amount of enzyme required to release 1 μmol of glucose or xylose per minute, respectively, under the established reaction conditions. Glucose and xylose were used as standards for cellulase and xylanase activities, respectively. The cellulase and xylanase activities were expressed in units per gram of dried and solid‐fermented substrate (U g^−1^).

### Statistical analyses

Data were checked for normality using Levene's test and transformed when required. One‐way ANOVA was used to assess the data and Tukey's test was used to discriminate significant differences among means (*P* < 0.05). All statistical analyses were conducted using IBM SPSS Statistics software, version 29 (IBM Corp., Armonk, NY, USA). Concerning the simplex centroid design, the substrate mixture that optimizes enzyme activity was determined using response optimization with Statgraphics Centurion XVI software (Statpoint Technologies, Inc., Warrenton, VA, USA) and Statistica 10 software (StatSoft, Inc., Tulsa, OK, USA).

## RESULTS AND DISCUSSION

### Screening of different enzyme‐producing fungi in SSF of frass

Several fungal strains were evaluated in the SSF of frass to select the strain with the highest chitinase and/or acid protease production. Regarding chitinases, enzymes produced by *Aspergillus niger* CECT 2088 achieved higher activity (34.4 ± 2.0 U g^−1^), followed by SSF with *Mucor circinelloides* CECT 2655 (18.35 ± 0.9 U g^−1^; Fig. 1(A)). The SSF of frass with the other fungi resulted in lower production of chitinases. Other studies have reported the use of insect‐derived substrates or chitin‐rich biomasses to produce chitinases through SSF. For example, the SSF of silkworm chrysalis was carried out using four strains of *Metarhizium anisopliae* (IBCB 167, IBCB 360, IBCB 384, IBCB 425; 26 °C, up to 13 days of duration), attaining higher endochitinase activity (7.14 U g^−1^) using the IBCB 360 strain, after 12 days and using 30% moisture.^38^ Wheat bran, shrimp shells, crab shells, molting of mealworms and puparium of black soldier fly were tested as substrates in SSF with *Talaromyces allahabadensis* Hi‐4 and *Talaromyces funiculosus* Hi‐5 (isolated from rotten *Hermetia illucens*), obtaining 46.8 U g^−1^ (Hi‐4 strain; 45.7% carbon source, 32 °C, 8.66 days) and 55.1 U g^−1^ (Hi‐5 strain; 62.3% carbon source, 28.3 °C, 5.77 days) of endochitinase activity using molting mealworms as substrate.^39^ Chitinases of fungal origin commonly belong to the glycosyl hydrolase (GH) 18 family, participating in the cell wall remodeling during fungal growth, use of chitin as energy source, among others.^40^ The GH 18 family is divided into three subgroups (A, B, and C).^41^ Subgroup C comprises chitinases linked to various carbohydrate‐binding modules that enhance the binding affinity of these enzymes to the substrates and have been discovered in Ascomycetes.^41^ Therefore, the higher chitinase activity observed in the *Aspergillus niger* CECT 2088 using frass as substrate in SSF may be due the ability of this species to produce type C chitinases. Nonetheless, future studies should focus on deepening the characterization of the enzymes produced by *Aspergillus niger* CECT 2088 during SSF.

**Figure 1:**
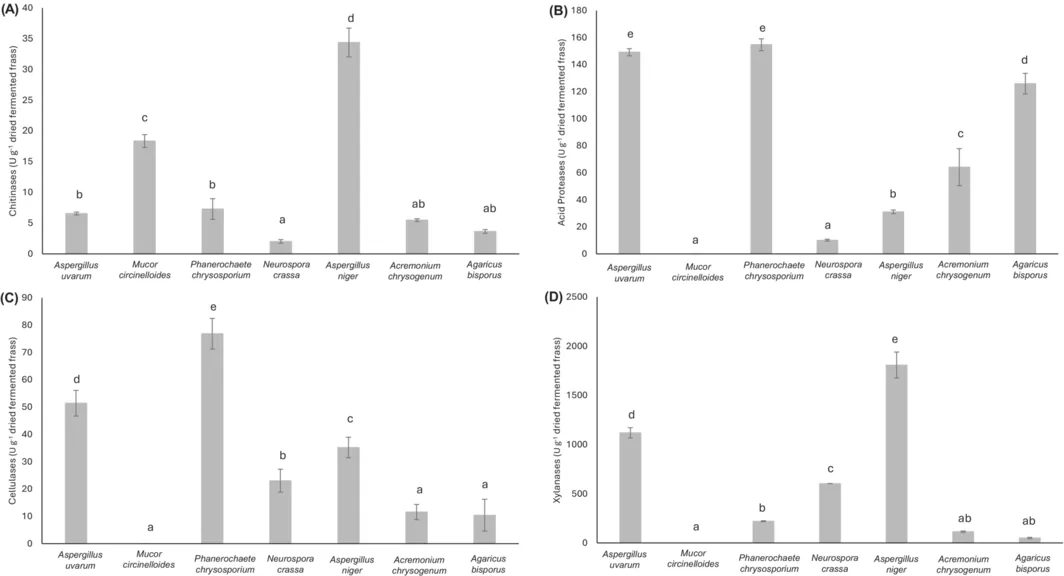
Screening of various fungal species in the solid‐state fermentation of frass for production of chitinases (A), proteases (B), cellulases (C), and xylanases (D) (U g^−1^ of dried fermented biomass). Regarding each enzyme, bars with different letters are statistically different (one‐way ANOVA, *P* < 0.05; Tukey's test).

Nonetheless, despite the continuing evolution of the use of insect by‐products in SSF, other biomasses are often used to produce chitinases, including lignocellulosic materials and marine discards. Other authors used shrimp shells as substrate in SSF with *Aspergillus terreus* and *Aspergillus niger* (30 °C, 5 days), resulting in 4.82 and 3.87 U mg^−1^ protein of chitinase activity, respectively.^42^ In another study, *Ganoderma lucidum* (white rot fungus) and fish scales were used as substrates (1:1, w/w proportion) in SSF with *Aspergillus terreus* SB3, attaining a chitinase activity of 496 U g^−1^ under optimized conditions (35 °C, 20 g DW of substrate, 4 days, 55% moisture, 0.25 g ammonium sulfate supplementation).^17^ *Aspergillus flavus* CFR10 and *Fusarium oxysporum* CFR8 isolated from soils resulted in 19.8 and 17.5 U g^−1^ of endochitinase production when solid fermenting wheat bran (55% moisture, 1% w/w chitin supplementation, 32 °C).^43^ The optimization of chitinase production using molokhia stems as substrate in SSF with *Aspergillus niger* (30 °C, 7 days) resulted in up to 12 841 U g^−1^ of chitinases (60 °C, pH 5, 0.25% of *p*‐nitrophenyl‐β‐d‐*N*‐acetyl glucosaminide).^44^ Similarly, in another study, optimum chitinase production (13 446 U g^−1^) was achieved using a combination of substrates (wheat, rice straw and sawdust) in SSF with *Aspergillus niger* EM77 (30 °C, 6 days).^44^ In this sense, since *Aspergillus niger* CECT 2088 resulted in higher chitinase activity, that work also focused on mixing frass with other products of insect origin, such as adult insects and chitin, in an attempt to improve protease and chitinase production.

Regarding acid proteases, *P. crysosporium* CECT 2777 (154.8 ± 4.4 U g^−1^) and *Aspergillus uvarum* MUM 08.01 (149.2 ± 2.7 U g^−1^) showed higher acid protease production, followed by *Agaricus bisporus* CECT 20291 (125.9 ± 7.6 U g^−1^; Fig. 1(B)). On the contrary, *Mucor circinelloides* CECT 2655 was the only fungus that did not demonstrate proteolytic activity. *Phanerochaete chrysosporium* is a basidiomycete which is highly efficient in degrading lignocellulosic substrates, possessing a wide range of carbohydrate‐active enzyme genes,^32^ which is in accordance with the present findings. Nonetheless, to the best of our knowledge, research on the use of insect by‐products in SSF for protease production is still at an early stage. In a previous work, frass from *Tenebrio molitor* was used as a substrate for SSF using different fungal strains (*Aspergillus sojae* CECT 2095, *Aspergillus uvarum* MUM 08.01, *Aspergillus oryzae* CECT 2094, *Trichoderma reesei* CECT 2414; 25 °C, 7 days), achieving 65 U g^−1^ (*Aspergillus uvarum* MUM 08.01), 45.3 U g^−1^ (*Aspergillus sojae* CECT 2095) and 42.7 U g^−1^ (*Aspergillus oryzae* CECT 2094 and *Trichoderma reesei* CECT 2414) of protease activity, which is around 30% and 34% lower, respectively, than the activity observed in this study using the same strain of *Aspergillus uvarum*.^12^ Indeed, this study follows the work of Muñoz‐Seijas *et al*.,^12^ attempting to expand the use of insect by‐products toward the production of chitinases and evaluating the capability of other fungal strains in producing both proteases and chitinases. Nonetheless, other authors have studied the production of proteases using different plant substrates in SSF (wood, wheat bran, soy flour, among others) and mainly using fungi of the genus *Aspergillus*. For example, 186.4, 152.4, and 118.8 U g^−1^ of protease activity was attained using wheat bran, soybean meal, or cottonseed meal, respectively, as substrates in SSF with *Aspergillus niger* (30 °C, 4 days).^45^ Similarly, higher protease (782 U g^−1^) production was achieved using *Aspergillus* spp. isolated from soil (Z1BL1) in SSF of a mixture of wheat bran and rice bran (30 °C, 6 days).^46^

Apart from *Aspergillus* spp., other promising fungal strains have been studied for their protease‐producing capabilities. Indeed, this study observed that *P. chrysosporium* CECT 2777 resulted in high protease activity, indicating the ability of this fungus to grow and produce extracellular enzymes using insect substrates. In another study, finger millet straw (*Eleusine coracana*) was solid fermented with *P. chrysosporium* (39 °C, 5 days) after steam treatment or supplemented with urea 4%, resulting in up to 2.64 and 2.40 U g^−1^ of proteases, respectively, which was much lower than the results observed herein.^47^ In this study, SSF using *N. crassa* CECT 2727 led to a residual production of acid proteases (10.1 ± 0.70 U g^−1^), indicating that this species may not be suitable for producing acidic proteases using frass as substrate. However, depending on the type of substrate, fungi may preferentially produce certain types of enzymes. For example, contrary to this study, solid‐fermented soybean bagasse (okara) with *N. crassa* (30 °C, 3 days) resulted in up to 1959.8 U g^−1^ of alkaline proteases, which is a much higher value than that obtained with the top protease producer of this study (*P. chrysosporium* CECT 2777).^19^

Furthermore, in this study, the production of cellulases and xylanases was also assessed after SSF of frass with the different fungal strains. Higher cellulase production (76.8 ± 7.00 U g^−1^) was achieved using *P. chrysosporium* CECT 2777, followed by *Aspergillus uvarum* MUM 08.01 (51.4 ± 4.82 U g^−1^) and *Aspergillus niger* CECT 2088 (35.2 ± 4.69 U g^−1^; Fig. 1(C)). Regarding xylanases, a higher activity (1809 ± 131.8 U g^−1^) was recorded using *Aspergillus niger* CECT 2088, followed by *Aspergillus uvarum* MUM 08.01 (1120 ± 42.07 U g^−1^; Fig. 1(D)). *Mucor circinelloides* CECT 2655 did not demonstrate either cellulase or xylanase activity. The high xylanase activity obtained in SSF of frass demonstrates the lignocellulosic richness of this substrate, mainly due to feed remaining that is deposited during insect rearing. In fact, the cellulase and xylanase activities detected in this study are similar to those achieved with lignocellulosic substrates, such as oil palm frond leaves,^48^ rice waste,^49^, ^50^ brewer's spent grain,^34^, ^51^ and wheat bran.^52^ Therefore, it is fair to assume that the enzymatic extract obtained after SSF of frass with *Aspergillus niger* CECT 2088 may have wide applicability, since it is enriched with xylanases and cellulases as well as chitinases and acid proteases.

### Optimization of protease and chitinase production using different insect by‐products as substrates in SSF

Following the selection of *Aspergillus niger* CECT 2088 as the most promising chitinase producer, a simplex centroid design was used to optimize the enzyme production using different insect by‐products: frass, adult insects, and/or chitin derived from an eco‐friendly processing of *Tenebrio molitor* adults with a deep eutectic solvent,^28^ separately or in combination. The model showed a good fit to the experimental data, achieving an *R* ^2^ of 0.9998 both for protease and chitinase production (Table 1). Chitinase production was maximized using either 100% frass or a mixture of 75% w/w frass and 25% w/w adult insects (Fig. 2(A)), while protease production was maximal using adult insects as the sole substrate in SSF (Fig. 2(B)). Other authors have resorted to simplex centroid designs to assess the optimal substrate mixtures for maximizing enzyme production. A simplex centroid design was used to study varied substrates in SSF with *Aspergillus niger* CECT 2915, showing that maximum protease production (>200 U g^−1^) was obtained using a substrate composed of equal parts of rapeseed cake and soybean cake.^53^ In another study, a simplex centroid design showed that protease production was maximal using exclusively wheat bran as substrate in SSF with *Aspergillus oryzae* LBA01 after 48 h (59.9 U g^−1^) and 72 h (43.3 U g^−1^), while mixing wheat bran with soybean or cottonseed meals achieved maximum activities (22.9 and 29.5 U g^−1^, respectively) after 48 h of SSF.^54^ In a follow‐up study, high protease production (262.8 U g^−1^) was also achieved after 48 h of SSF with *Aspergillus niger* LBA 02 using a substrate composed of equal parts of wheat bran and soybean meal.^55^ Therefore, a mixture of substrates may be beneficial for enzyme production, allowing the joint reutilization of different by‐products and contributing to a more efficient use of residual biomass.

**Table 1:** Regression coefficients of the simplex centroid design used in the optimization of chitinase and protease production in SSF of frass and *Tenebrio molitor* adults, respectively, with *Aspergillus niger* CECT 2088

Regression coefficient	Chitinase	Protease
*X*_1_	0	0
*X*_2_	9.92*	248.75*
*X*_3_	34.39*	31.08*
*X*_1_ *X*_2_	9.08*	−401.86*
*X*_1_ *X*_3_	0.06	−41.8**
*X*_2_ *X*_3_	32.22*	−502.62*
*X*_1_ *X*_2_ *X*_3_	−3.40866	810.668*
*R* ^2^	1	1
Adjusted *R* ^2^	0.9998	0.9998

R^2^, coefficient of determination. Asterisks indicate *P < 0.01, **P < 0.05.

**Figure 2:**
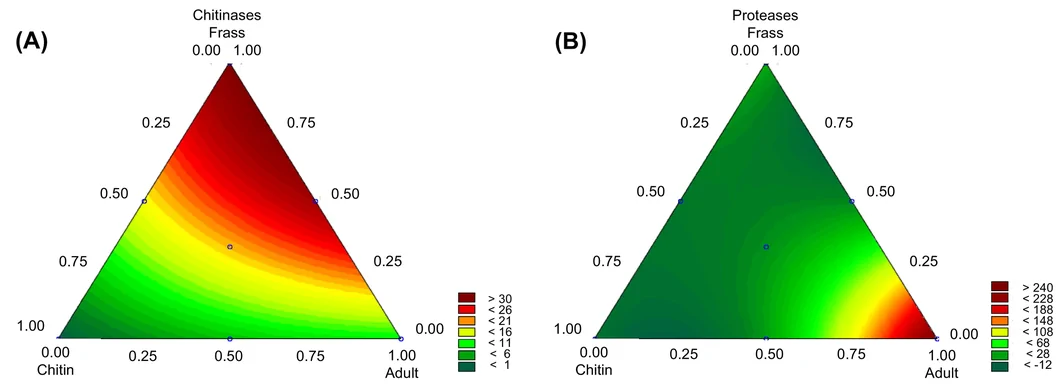
Contour plots for dependent variables of simplex centroid design: (A) for chitinases; and (B) for proteases.

The maximum protease activity achieved using solely adult insects as substrate in SSF with *Aspergillus niger* CECT 2088 may be due to the high protein content observed in adult insects (47.9% DW) compared with that observed in frass (24.3% DW).^12^ Additionally, adult insects present a lower carbon‐to‐nitrogen ratio (5.3) than frass (8.77),^12^ which may benefit protease production, as was also observed by de Castro *et al*.^45^ These authors observed that lower carbon‐to‐nitrogen ratios and the high protein content of soybean meal (49.2%) benefited protease production (152.4 U g^−1^) during SSF of soybean meal with *Aspergillus niger*. Similarly, since higher chitin content is found in adult insects (22.7% DW) compared with frass (7.40% DW), higher chitinase activity would be expected when using adult insects as substrate in SSF with *Aspergillus niger* CECT 2088, which was not observed. However, it is important to consider that the chitin present in adult insects is tightly bound to the protein fraction, which is evidenced by the need to use highly concentrated solvents and harsh conditions to separate the components.^56^ Therefore, the fungus seemed to have limited access to the chitin present in adult insects, which may have hindered chitinase production. Concurrently, the lower chitin content of frass and the presence of other nutrients seems to be sufficient to induce chitinase production by *Aspergillus niger* CECT 2088 without hampering fungal growth. In fact, in this work, *Aspergillus niger* CECT 2088 produced high xylanase (1809 U g^−1^) and cellulase (76.8 U g^−1^) activities, indicating the ability of the fungus to use the lignocellulosic fraction to grow, which concomitantly may have benefited the production of chitinases.

In this study, the addition of chitin extracted after treating adult insects with deep eutectic solvents to frass or adult insects did not improve chitinase and protease production by *Aspergillus niger* CECT 2088, which was also confirmed by the *P*‐values of the regression coefficients (Table 1). Colloidal chitin is considered the best inducer of chitinase production by fungi.^25^ However, contrary to what was expected, using chitin of insect origin in this work did not improve enzyme production. This chitin was directly obtained from fractioning adult insects using deep eutectic solvents and may require further purification steps before it is suitable to promote fungal growth via SSF, while the colloidal chitin often used is a purified and commercial substrate. Additionally, residual deep eutectic solvents may have remained in the chitin extracted from *Tenebrio molitor* adults, hindering fungal growth and the concomitant production of chitinases. Indeed, betaine‐based compounds may exert antimicrobial effects for a wide range of bacteria and fungi,^57^ which might have contributed to the low chitinase and protease production. Besides, the possible presence of residual betaine and urea in the chitin may hinder enzyme production during SSF since urea may denaturate protein, hindering the growth of fungi and thus chitinase production. Although the presence of betaine may counteract the adverse effects of urea and stabilize proteins, the presence of low concentrations of betaine and urea in chitin along with water to maintain the moisture of SSF at 750 g kg^−1^ may promote stronger interactions between urea and water instead between urea and betaine, boosting the denaturating feature of urea and compromising enzyme activity during SSF.^58^ Nonetheless, resorting to experimental designs allows fine‐tuning of the mixture and composition of substrates to maximize enzyme production, while envisioning whether the simultaneous utilization of disposal biomass is possible. Hence, since both frass and adult insects resulted in higher chitinase and protease production when used separately, the kinetics of enzyme production was also studied during SSF of these biomasses.

### Kinetics of protease and chitinase production during SSF of frass and adult insects

After selecting *Aspergillus niger* CECT 2088 as the most suitable enzyme producer in SSF of frass, the kinetics of chitinase and protease production were studied using frass and adult insects, respectively. Highest chitinase production (53.3 ± 4.53 U g^−1^) was attained on the 11th day of SSF of frass, equivalent to a 19.2% increase in enzymatic activity with respect to the initial screening of fungal strains, which was carried out for 7 days (Fig. 3(A)). However, the chitinases decreased after the 12th day, probably due to nutritional depletion and/or the release of toxic metabolites during SSF.^17^ Also, the higher chitinase activity observed at the 11th day may also be related to an initial adaptation of the fungus to the substrate, since the early production of other important enzymes, such as carbohydrases and proteases, seem to be important to promote a more efficient use of the substrate while, in a more advanced phase of SSF, chitinases start to be formed to meet other biological functions besides nutrient uptake. Chitinases are involved in fungal growth and autolysis, degrading microbial cell walls and promoting hyphal growth and expansion.^25^, ^59^ It is worth noting that fungi growing in varied lignocellulosic substrates often start producing carbohydrases, such as xylanase and cellulases, to help release the nutrients entrapped in the rigid lignocellulosic matrix, progressing to producing other important enzymes, including proteases and lipases, among others.^60^ Therefore, the importance of chitinases goes beyond hydrolyzing the solid substrate for nutrient absorption, which may explain why higher activities can be often observed in an advanced phase of SSF. The study of kinetics of chitinase production during SSF is still underdeveloped and, to the best of our knowledge, there are no studies on this subject using chitinases originating from SSF of insect‐based substrates. However, other authors have studied the production of chitinases using other substrates in SSF. The SSF of wheat bran with *Trichoderma koningiopsis* UFSMQ40 (30% w/w corn steep liquor, 50% moisture, 10% w/w chitin, 1 inoculum disk) reached maximum chitinase activity (10.76 U g^−1^) on the 3rd day, subsequently decreasing.^61^ Therefore, although literature is scarce, extending the SSF up to the 11th day increased chitinase production comparatively to the 7‐day SSF, confirming the importance of fine‐tuning the duration of fermentation to achieve maximum enzyme activity. Nonetheless, aiming to increase the cost‐effectiveness of chitinase production and according to the quantity required of this enzymatic extract, some operative parameters could be considered and adjusted to decrease cost and maximize production, such as adjusting the fermentation time and inoculum size, among others.

**Figure 3:**
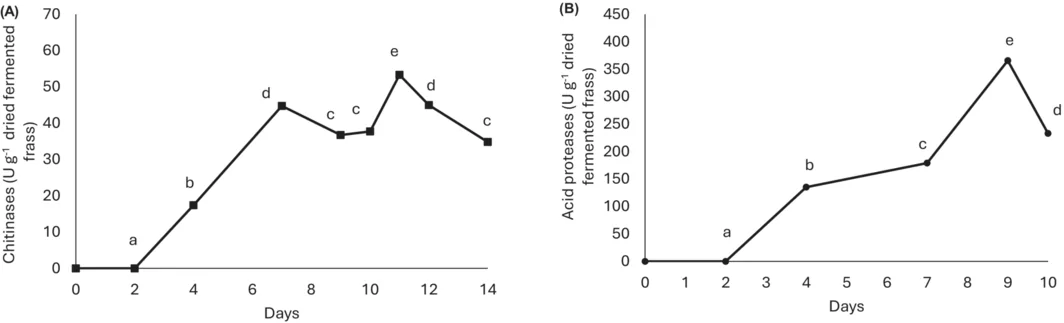
Kinetics of chitinase (A) and protease (B) production during the solid‐state fermentation of frass and adult insects with *Aspergillus niger* CECT 2088, respectively. Different letters indicate differences between sampling times (one‐way ANOVA, *P* < 0.05; Tukey's test).

Regarding using adult insects as substrate in SSF, acid proteases started being produced on day 4 (135 ± 7.50 U g^−1^), achieving maximum activity at the 9th day (366 ± 15.8 U g^−1^), which represents a twofold increase compared to the activity observed on the 7th day of SSF (179 ± 11.0 U g^−1^; Fig. 3(B)). A negative trend in protease activity was observed after the 9th day, registering a 36.3% reduction in activity compared to the maximum value. Another study observed that maximum protease activity (specific activity of 1.14 U mg^−1^) was attained on the 3rd day of SSF of wheat bran with *Rhizomucor miehei* Rm4 isolate, under optimized conditions of 41.1 °C, pH 6.31, 80% moisture and 1.33% casein supplementation.^62^ Similarly, other authors observed maximum protease activity (2331 U g^−1^) after 33 h of SSF of a mixture of defatted soybean meal and wheat bran (6:4 w/w) with *Aspergillus oryzae* 3.042.^63^ The lower protease activity observed in this study comparative to the values observed in the literature may be related to the tight bond that exists between the protein and chitin in adult insects, which may decrease the accessibility of *Aspergillus niger* CECT 2088 to the protein and may hinder the production of proteases. Importantly, chitin present in insect cuticles is embedded in proteins forming a very organized and thick structure and is often mineralized to resist mechanic and thermal stress.^64^ Therefore, this complex structure may have decreased the accessibility of the protein to *Aspergillus niger* CECT 2088 and concomitantly compromise the production of proteases. Nonetheless, it is important to point out that no nutrients or salts were supplemented to the adult insects in this study, which should be considered in future studies attempting to maximize enzyme production.

### Tray reactor experiments in the SSF for chitinase and protease production

In this study, the quantity of frass or adult insects used in SSF increased to 50 and 100 g DW, respectively, assessing the effect on chitinase and protease production, under optimized conditions. Increasing the quantity of frass in SSF up to 50 g did not decrease chitinase production compared to using 10 g DW, while the activity decreased to 34.2 U g^−1^ when 100 g DW of frass was used (Fig. 4(A)). Regarding the SSF of adult insects, protease production decreased by 70% and 44% when 50 and 100 g of substrate were used, respectively (Fig. 4(B)). Other studies have focused on increasing the amount of substrate used in SSF to produce enzymes, but the use of insect by‐products is still vastly underexplored, especially for chitinase production. Contrary to this study, Muñoz‐Seijas *et al*. (2024)^12^ increased the amount of substrate (mixture of insect frass and BSG, 1:1 w/w) up to 500 g DW in SSF with *Aspergillus uvarum* MUM 08.01 and observed an almost fourfold increase (202 U g^−1^) in protease activity. Nonetheless, the bioreactor design greatly affects the efficiency of producing the desired compounds using fungi. For example, a double SSF was carried out in 1 kg of a substrate composed of palm kernel cake and plan‐pressed fiber (70:30 ratio) with *Aspergillus oryzae* using a 15 L rotating drum bioreactor and obtained 136.7 U g^−1^ of protease activity – a lower value compared to when using 5 g of substrate in the SSF (208.4 U g^−1^; 55% moisture, 52 h).^65^ Conversely, other authors observed that carrying out SSF with 400 g of babassu cake with *Aspergillus awamori* IOC‐3914 using a cylindrical fixed‐bed bioreactor with forced aeration potentiated the production of varied enzymes, including proteases (31.8 U g^−1^; 168 h).^66^

**Figure 4:**
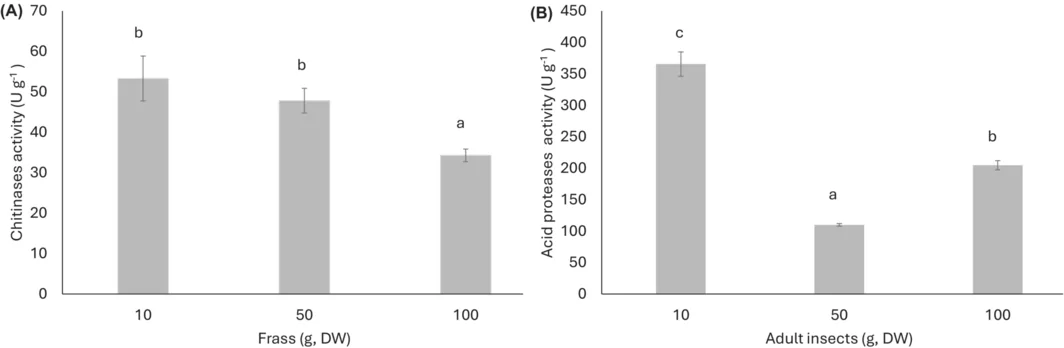
Scale‐up of solid‐state fermentation of frass and adult insects for chitinase (A) and protease (B) production, respectively, using *A. niger* CECT 2088. Bars with different letters are statistically different (one‐way ANOVA, *P* < 0.05; Tukey's test).

Incrementing the quantity of substrate in SSF presents many challenges associated with the dissipation of metabolic heat generated within the substrate, water evaporation, and heterogeneity of fermentation.^67^ In this study, tray‐type reactors used in SSF with either 50 g DW or 100 g DW of substrate with *Aspergillus niger* CECT 2088 guaranteed a layer height between 2 and 2.5 cm. Nonetheless, using 100 g DW of substrate decreased chitinase production, and using 50 and 100 g of adult insects decreased protease activity, which may be due to substrate overheating during fermentation and/or humidity reduction. Therefore, this study should be considered in relation to future studies centering on testing higher solid loads of insect‐origin substrates in SSF, as well as considering distinct airflow rates to aid in maintaining an appropriate temperature within the substrate bed without drying the biomass and/or harming the fungal strains.^68^

## CONCLUSIONS

The use of insect frass and adult insects as substrates in SSF with filamentous fungi is an efficient strategy to reutilize these inherent and abundant by‐products toward the production of chitinases and proteases, respectively. *Aspergillus niger* CECT 2088 was the most efficient fungal strain in producing chitinases after 11 days SSF using frass, while high protease activity was attained after 9 days of SSF using adult insects as substrate. The simplex centroid design confirmed that chitinase and protease production was favored when using solely frass or adult insects, respectively. Nonetheless, future studies should consider testing varied mixtures of frass with adult insects, since a synergic effect between both substrates was observed regarding chitinase production. Also, because scaling up the SSF of both substrates up to 100 g decreased enzyme production, future work should focus on studying the possible adaptation of SSF conditions to tray reactors to ensure high enzyme production when using higher quantities of insect by‐products in SSF.

Therefore, the present work demonstrates the importance of generating high‐value enzymes via SSF using residual biomass from a growing insect industry. Future studies should focus on characterizing the chitinases and proteases obtained by SSF of insect residues, assessing their suitability for use in important industrial processes, ultimately contributing to a circular economy and zero‐waste policies.

## Data Availability

The data that support the findings of this study are available from the corresponding author upon reasonable request.
